# Relationship between psychosocial problems and satisfaction with GP communication in German primary care practices: a structural equation model based on the cross-sectional GPCare-1 patient study

**DOI:** 10.1136/bmjopen-2024-095489

**Published:** 2025-05-07

**Authors:** Juliane Sachschal, Thomas Welchowski, Luisa Offenberg, Maja Oberholz, Boris Gavrilov, Nur Ikar, Carmen Hunzelar, Florian Bockheim, Joana Paños-Willuhn, Birgitta Weltermann

**Affiliations:** 1 Institute for General Practice and Family Medicine, University Hospital Bonn, Bonn, Germany; 2 Department of Psychology, University of Zurich, Zürich, Switzerland; 3 Institute for Medical Biometry, Informatics and Epidemiology, University of Bonn, Bonn, Germany

**Keywords:** PUBLIC HEALTH, Primary Health Care, SOCIAL MEDICINE

## Abstract

**Objectives:**

This study examined the relationship between primary care patients’ psychosocial problems, other patient characteristics that are associated with satisfaction with overall care and satisfaction with general practitioner (GP) communication.

**Design:**

A cross-sectional survey was conducted. Patients filled an anonymous two-page questionnaire on various socio-demographic, medical characteristics and their satisfaction with GP communication. Structural equation modelling evaluated associations of various patient characteristics, including psychosocial problems with GP communication.

**Setting:**

General practices in Germany.

**Participants:**

A total of 813 patients from 12 GP practices participated. The survey was conducted in summer 2020 during a COVID-19 lockdown.

**Results:**

The estimated response rate was 24.1%. The prevalence of psychosocial problems in the sample was 30%. The three most frequent problems were excessive stress at work (19%), financial problems/debts (9%) and loneliness (8%). Most patients agreed that their GP takes their problems seriously (71%), feeling comfortable talking about sensitive things (66%), having enough space in communication (62%) and being asked by their GP about personal strains (53%). Higher social support, preference to solve one’s problem without GP help, higher age and better health status predicted more satisfaction with physician–patient communication, while the number of psychosocial problems, gender, years with physician, chronic stress and depression had no influence. According to the Bentler Comparative Fit Index, the pooled structural equation model had a 97.6% better fit than the corresponding model without covariate effects.

Higher social support, preference to solve one’s problem without GP help, higher age and better health status but not the number of psychosocial problems predicted more satisfaction with physician–patient communication.

**Conclusions:**

GPs should be aware of the high occurrence of patients’ psychosocial problems and actively address patients’ social support and self-management preferences which influence patients’ satisfaction with GP communication.

**Trial registration number:**

The General Practice Care-1 study was registered in the German Clinical Trials Register (DRKS00022330).

**STRENGTHS AND LIMITATIONS OF THIS STUDY**

- The study examines the prevalence of psychosocial problems and satisfaction with general practitioner (GP) communication in primary care patients from a patient rather than a provider’s perspective.
- The study recruited a large sample of German primary care patients (n=813) from different socio-economic backgrounds, stratified by region.
- Structural equation modelling was used to estimate a multiple parameter model that predicted patient satisfaction with GP communication.
- The patient’s response rate could only be estimated; the estimated response rate is low (24.1%).
- The patient sample is not representative, but in some characteristics similar to national comparative values.

## Background

 Psychosocial aspects of life influence morbidity and mortality.^1^ For example, lower socio-economic status and lower income are related to shorter life expectancy and poorer quality of life.^2^,^4^ Also, chronic stress, social isolation and financial problems are associated with a higher prevalence of adverse outcomes related to cardiovascular diseases,^5 6^ cerebrovascular diseases, hypertension^7^,^9^ and cancer.^10^,^12^ It has further been shown that considering patients’ contextual factors, such as financial or transportation problems, can play an important role in improving patient outcomes and decreasing healthcare costs.^13^,^15^ Aiming to improve health outcomes for these populations, general practitioners (GPs) can play an important role due to their personal relationship to patients from various backgrounds with the potential to address psychosocial problems.^15^ However, contextual factors are not always identified and addressed in primary care.

While collaborative structures of physicians and social workers are being implemented in a few countries like Great Britain and Ireland,^16^ studies from other settings report that patients’ psychosocial needs are often not identified^17^ and not addressed by GPs.^18^,^20^ In line with this, GPs reported lower prevalence rates of patients’ psychosocial problems such as financial difficulties, personal stress or unemployment than their patients.^21^ A study from the Danish primary care setting showed that GPs typically address biological and psychological issues, but feel uncomfortable addressing patients’ social needs due to a lack of training and knowledge of resources.^22^ A study from Norway shows that only 17% of the consultations were influenced by the GPs’ knowledge of their patients’ social problems.^23^

Adequate GP communication is the basis to address psychosocial problems in consultations and is shown to be the strongest driver of patient satisfaction with primary care.^24^ However, factors influencing satisfaction with GP communication, and especially the association between psychosocial problems and satisfaction with GP communication, have not been studied widely. Several studies examined patient-related factors that influence satisfaction with GP care. For example, lower age,^25 26^ poorer self-reported mental health,^27 28^ lower physical health status^26 27 29 30^ and lower perceived social support^27 31^ were associated with less patient satisfaction with care. Yet, it has not been systematically investigated how these factors influence patients’ satisfaction with GP communication.

A study by Gulbrandsen and colleagues^21^ highlights the importance of communication in primary care, showing that patients disclosed less than half of their reported problems to their GP. Using data from more than 800 patients from the German General Practice Care-1 (GPCare-1) patient survey, this study addresses the relationship between patients’ psychosocial problems, other patient characteristics associated with satisfaction with overall care and the satisfaction with GP communication.

## Methods

The GPCare-1 study is a cross-sectional study conducted by the Institute of Family Medicine and General Practice, University of Bonn, Germany. It examined the following three aspects:

- The prevalence of psychosocial problems in adult GP patients from practices of our teaching practice research network,
- Patients’ satisfaction with GP communication.
- The associations between psychosocial problems, patient-reported characteristics and patients’ satisfaction with GP communication.

### Design, settings and patients

#### Practice recruitment

The study was conducted in 12 primary care teaching practices, which are affiliated with the Institute of General Practice and Family Medicine, University of Bonn and University Hospital Bonn. Practices were selected based on different socio-demographic regional characteristics (eg, age structure, population density, proportion of migrants) to ensure a coverage of differing population groups. The survey was conducted between June and August 2020 which happened to be during the second COVID-19 lockdown in Germany. As practices were too busy during the COVID-19 pandemic, the response rate was calculated based on average data of patients per practice from a public German database^32^ and a documented 40% reduction in patient volume in GP practices.^33^ The calculated response rate was 24.1. The targeted sample size was 1000 patients. The goal could not be reached due to slower recruitment in primary care practices during the COVID-19 pandemic and its lockdowns.

#### Participants’ recruitment

All adult patients who visited one of the practices during the survey period and were able to fill in a questionnaire in the waiting room were eligible. Reception clerks offered the study material which comprised an information letter and a two-page questionnaire in different languages (German, English, Arabic, Turkish). It took about 10 min to fill in the questionnaire. Completed questionnaires were dropped into a locked ‘post box’ in the practice using a sealed envelope. Patients were informed about the anonymity of the survey, their voluntary participation and the aim of the study both verbally and in writing. By participating, patients declared their consent.

### Measures

The GPCare-1 questionnaire comprised a total of 48 questions (for more details, see online supplemental material 1). It integrated existing questions from standardised surveys as well as self-developed items. Questions addressing patients’ socio-demographic characteristics, health and psychosocial characteristics were mainly derived from the DEGS1 questionnaire used by the Robert-Koch-Institute for the national health monitoring system (DEGS1: German Health Interview and Examination Survey for Adults).^34^ Additional four questions addressed patients’ experiences in communicating with their GP. The questionnaire was piloted with 40 individuals from the German general population with minor adjustments thereafter. The following aspects were included:

The GPCare-1 questionnaire comprised a total of 48 questions (see online supplemental material 1). It integrated existing questions from standardised surveys as well as self-developed items. Questions addressing patients’ socio-demographic characteristics, health and psychosocial characteristics were mainly derived from the DEGS1 questionnaire used by the Robert-Koch-Institute for the national health monitoring system (DEGS1 stands for the German Health Interview and Examination Survey for Adults) .^34^ Additional four questions addressed patients’ experiences in communicating with their GP. The questionnaire was piloted with 40 individuals from the German general population with minor adjustments thereafter. The following aspects were included:

- *Socio-demographic characteristics*: Age and gender (male/female/divers).
- *Education:* Current profession, work sector, highest educational level (low=did not complete any education/secondary school up to ninth grade/secondary school up to tenth grade, middle=high school (A-levels)/vocational school; high=university degree), current occupational status and monthly household net income.
- *Living conditions*: Relationship status, informal caregiving, migration background and household size.
- *Social support* was measured with the Oslo scale.^35^ It categorises participants’ perceived availability of social support into low, medium and high.
- *Health-related factors (physical*): Time with the GP as a patient, general health status, specific health problems (eg, diabetes, high blood pressure) and self-management style (preference to solve problems on one’s own).
- *Depressive symptoms* were measured with the PHQ-2 of the Patient Health Questionnaire (PHQ).^36^ The PHQ-2 is a brief screening instrument to assess the severity of depressive symptoms. It consists of two items that ask about depressive symptoms over a period of the last 14 days. Answer possibilities range from 0 (never) to 3 (almost every day), with a maximum of six points.
- *C*hronic stress (last 3 months) was measured with the 12-item screening tool TICS-SSCS (Trier Inventory for Chronic Stress).^37^ A sum score was calculated and classified into three categories: low (0–11), middle (12–22) and high (22–28).
- *Psychosocial problems:* Patients were asked whether they were currently affected by any of the following problems: excessive stress at work, loss of job/unemployment, feeling of loneliness, taking care of a relative or (family) friend (informal caregiving), financial problems/debts that are difficult to negotiate, death of a partner, physical attacks, psychologically hurting actions or threats, sexual harassment and assaults.
- *Satisfaction with GP communication:* Four items addressed physician–patient communication with previous GP contacts based on existing instruments. Participants were asked for their agreement to various statements using a five-point Likert scale (strongly disagree to strongly agree). A sum score was calculated, with higher scores indicating more satisfaction with GP communication. The following instruments were considered in the development of these items: the Medical Interview Satisfaction Scale,^38^ the Patient Request Form,^39^ the Patient-Doctor Relationship Questionnaire^40^ and the Patient Reaction Assessment Instruments.^41^

### Data analysis

The patient sample was described using descriptive statistics and frequencies. Per patient, the number and kind of problems currently burdening was summed (0, 1–2, 3–4, 5+). Differences in prevalence were investigated using χ^2^ test. Missing rates are displayed in brackets behind the respective item. All percentages are displayed as valid per cent.

Associations between communication and satisfaction, as well as their dependencies, were jointly estimated by a structural equation model (SEM). The latent continuous endogenous variable satisfaction with GP communication represents one dimension of patient satisfaction with quality of care, including perceived consideration for the patient^42^ and emotional support.^39^ Communication quality is a subdimension of the interpersonal qualities of a GP.^43^ The SEM consists of a structural component that is represented on the left side of the latent variable in figure 2 and a measurement model displayed on the right side of the latent variable. Outgoing arrows represent independent variables and ingoing arrows dependent variables. All variables used in the SEM were assumed to be observations from a continuous scale. This includes the summary variables derived from multiple itms, such as the sum of PHQ items, sum of TICS-SCSS (Trier Inventory for Chronic Stress) items and the number of impairments.

The structural component part can be interpreted analogously to a linear regression framework.^44^ All observed items on the left side (age, gender, social support, time with the GP, depression score, chronic stress, number of current psychosocial problems, health status and communication preference) correspond to independent covariates, and the latent variable satisfaction with GP communication is the dependent response variable. The latent variable is assumed to be continuous and normally distributed conditional on the items. Each path represents the effect of the specific item on the latent variable. Due to the continuous scale of observed variables, each coefficient represents the linear effect of the covariate on satisfaction with GP communication if the covariate would be increased by one unit, given all other covariates stay constant.

In the measurement model part, the observed items (communication 1–4) are responses that are explained by the latent variable satisfaction with GP communication analogue to factor analysis.^45^ The coefficient of personal strains was restricted to 1 due to identifiability constraints. Each path represents factor loadings that can be interpreted as regression coefficients between covariate satisfaction with GP communication and each item. Values near 1 are an indication of good correspondence between the construct satisfaction with GP communication and measured items (eg, comfortableness, problem perception).

In the model, the latent variable depends on the observed items age, gender, social support, time with the GP, depression score, chronic stress, number of current psychosocial problems, health status and communication preference. Satisfaction was measured by observed items communication 1–4.

Missing values were imputed by multiple imputation by chained equations,^46^ with 25 iterations and repetitions. Continuous covariates (eg, age, Oslo score) were imputed by predictive mean matching, nominal covariates (eg, gender) were imputed by multinomial regression^47^ and ordinal covariates (eg, health status) were imputed by proportional odds models.^48^ For each multiple imputed data set, a SEM was estimated.^49^ All items were assumed to be ordinal representations of continuous scales. Norman^50^ points out that many previous studies show the robustness of Likert scales to parametric assumption violations and that parametric tests can be applied for Likert scales. According to the recommendations of Kline,^49^ we report several pooled SEM goodness-of-fit statistics. Among those is the χ^2^ test statistic of the SEM, which is an omnibus test with the null hypothesis that all coefficients are zero. The Bentler Comparative Fit Index^51^ compares the model with the previous null hypothesis model and calculates the relative difference. Steiger–Lind Root Mean Square Error of Approximation^52^ and the standardised root mean square residual both compare the estimated values of the SEM with the observed data. In the former, values below 0.05 and for the latter measure values below 0.08 indicate a good model fit.^53^ We further conducted several sensitivity analyses (cluster analysis, complete case analysis). The cluster information did not systematically improve the fit of the SEM to the data. The complete case analysis did not indicate systematic differences between the complete case SEM and the multiple imputed version. Results of both analyses can be found in the online supplemental file 2.

IBM SPSS Statistics 25 for Windows was used^54^ for the first part of the analyses. The SEM was conducted with statistical software R V.4.2.2.^55^ The SEM was estimated using default settings in R-package lavaan V.0.6-15^56^ by maximum likelihood method. Variances of the latent variable and their measurement variables were not fixed and estimated from the data. The model consists of 17 parameters (structural part 10 parameters, measurement part 3 parameters and variance estimation 4 parameters). The sample size to estimated parameters ratio is 47.71 which is more than double the recommended minimum ratio of 20 in Kline.^49^ In this work, p values <0.05 are considered significant.

### Patient and public involvement

GPs were involved in the planning and design of the study design and the questionnaires. 40 persons from the general public were involved in pretesting the questionnaires. Patients were involved as participants in the conduct of the study. The findings will be presented to and discussed with GPs and patients from our practice and research network.

## Results

### Sample description: socio-demographic and health characteristics

The GPCare-1 data set included 813 adult GP patients. Characteristics are displayed in table 1. The mean age was 52 years (range 18–91 years). The sample included about 59% females. 25% of the participants had a migration background. More than 60% of the participants were with their GP for more than 5 years (65%). The majority of participants reported middle or high social support (middle: 52%; high: 28%), while 21% of the participants reported low social support. Almost one-third of the patients indicated excessive stress (19%), and 42% reported bad general health. The most frequent health problems of the participants were back and/or joint complaints (55%), high blood pressure (36%) and sleeping disorders (31%).

**Table 1:** Sample description (n=813)

	N	%
Gender (13*)		
Female	474	59.3
Male	337	41.4
Diverse	2	0.3
Age, mean, SD (13*)	51.61	18.7
Age groups (13*)		
18–39	243	30.4
40–59	266	33.3
60–69	130	16.3
70–79	103	12.9
80+	58	7.2
Chosen questionnaire language (0*)		
German	761	93.6
Other	52	6.4
Migration background (36*)	194	25.0
Education (23*)		
Low	247	31.3
Middle	336	42.5
High	190	24.1
Other	17	2.2
Social support (48*)		
Low	157	20.5
Middle	398	52.0
High	210	27.5
General health status (subjective) (20*)		
Moderate, bad, very bad	333	42.0
Excellent, very good, good	460	58.0
Health problems (38*)		
Back/joint complaints	428	55.2
High blood pressure	282	36.4
Sleeping disorders	240	31
Migraine	90	11.6
Coronary artery disease	82	10.6
Chronic obstructive pulmonary disease	64	8.3
Depressive symptoms (Patient Health Questionnaire 2), mean, SD (97*)	1.75	1.62
Chronic stress (TICS-SSCS*), mean, SD (125*)	17.01	10.4
Low	223	27.4
Medium	260	32.0
High	205	25.2
Years with general practitioner (26*)		
<3	150	18.9
3–5	122	15.5
>5	515	65.4
Number of current psychosocial problems per patient, categorised (34*)		
None	535	68.7
1–2	199	25.5
3–4	36	4.6
5+	9	1.2
Satisfaction with general practitioner communication, mean, SD	15.19	4.19

*TICS-SSCS: Trier Inventory for Chronic Stress (validated survey instrument)

* Missing values are described after each variable (N).

70% of the patients did not report any psychosocial problems, while about a fourth (25%) reported 1–2 problems, 4% 3–4problems and about 1% had 5 or more challenges. The most reported psychosocial problems by GP patients were stress at work (19%), feeling of loneliness (9%) and financial difficulties (7%). Table 2 displays how many of the patients reported psychosocial problems in GP practices and self-management preferences of those patients who reported at least one current psychosocial problem.

**Table 2:** General Practice Care-1 study: percentage of patients who indicated current psychosocial problems (multiple select answer format) and percentage of those who have current social problems that would rather like support by their general practitioner (disagree/rather disagree to wanting to solve problems without general practitioner)

Type of psychosocial problem	Reported current psychosocial problems (%)	Of those reported current problems would like help (%)
Excessive stress at work	19.2	22.1
Feeling of loneliness	9.0	17.2
Financial problems/debts	7.7	14.3
Taking care of a relative or (family) friend	5.3	28.2
Loss of job/unemployment	5.3	21.6
Psychological damaging actions/threats	4.7	21.2
Death of a partner	2.1	33.3
Sexual harassment	1.8	28.6
Physical attacks	1.6	27.3
Sexual assaults	1.1	42.9

### Physician–patient communication

**Figure 1:**
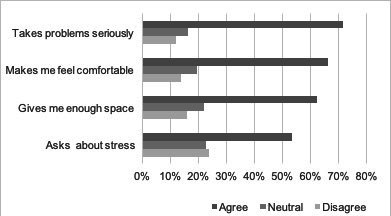
General Practice Care-1 study: patients’ satisfaction with general practitioner communication (in %).

More than half of the patients agreed or agreed strongly to each of the four communication statements. In detail, 71% agreed that “the doctor takes my problems seriously”, 66% reported being “made feeling comfortable when talking about sensitive things”, 62% were “given enough space to describe personal strains” and 53% were “asked about stress caused by personal strains”. For details, see figure 1.

### Modelling satisfaction with GP communication

**Figure 2:**
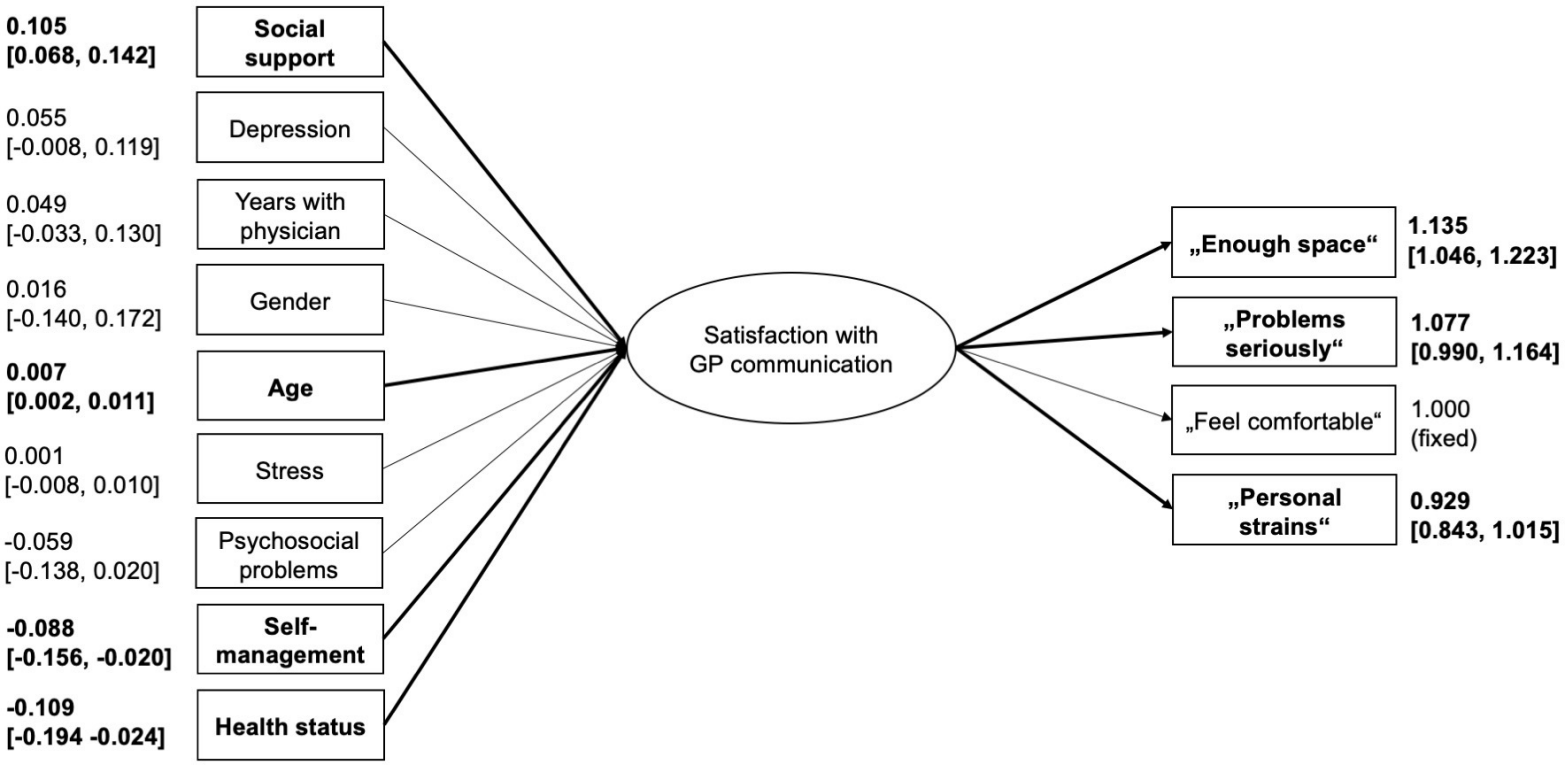
Structural equation model with endogenous continuous latent variable satisfaction, which depends on the observed items on the left and was measured by the variables on the right. GP, general practitioner.

The SEM was estimated as described in the section ‘Data analysis’. The estimated SEM parameters are shown in figure 2. The variables social support, health status and self-management preference, and age predicted the latent variable satisfaction, suggesting that higher age, more social support, better health status and the preference to not solve problems on their own were associated with higher satisfaction with GP communication.

## Discussion

The present study examined psychosocial problems in GP practices, patients’ satisfaction with GP communication and the relationship between psychosocial problems, other patient-related variables and patients’ satisfaction with GP communication. About every third primary care patient reported at least one current psychosocial problem, with the most common being stress at work (19%), loneliness (9%) and financial problems (8%). Generally, patients were satisfied with GP communication, and most patients did not explicitly prefer help from their GP to solve their problems. Higher social support, preference to solve one’s problem without GP help, higher age and better health status but not the number of psychosocial problems predicted more satisfaction with physician–patient communication. To the authors’ knowledge, this is the first study that examined psychosocial problems and patient–physician communication in a large primary care patient sample in Germany. GPs should be aware of the presence of current social support and patients’ self-management preference as important factors associated with patients’ satisfaction with GP communication.

The study assessed the prevalence of psychosocial problems in German primary care patients from a patient perspective. Selected GP practices in different regions were chosen in order to reach a variety of patients from different social backgrounds. The nature of psychosocial problems reported by GP patients in this study is in line with those reported by German GPs.^19^ The reported prevalence rates in this sample were similar to those reported in a study of GP patients in Norway, for example, stressful working conditions (25%) or loneliness (7%).^20^ The reported psychosocial problems seem to be more prevalent than reported by GPs, who indicated that psychosocial problems play a role in their consultations at least three times per week.^19^ This finding, in turn, is in line with Bikson *et al*^57^ and Gulbrandsen *et al,*^20^ who found that the prevalence of psychosocial problems in GP practices was higher when assessed through patients compared with GPs. Furthermore, the prevalence of some self-reported psychosocial problems in GP patients found in this study seems higher than in the German general population. For example, only 11% of the German population reported chronic stress in the DEGS study,^34^ which is lower than the percentage of patients who reported currently being burdened by stress at work in this study. The prevalence of loneliness in this study was similar (with 9%) to the one reported in the city of Leipzig, where 12% of the population reported being lonely in 2011.^58^

SEM was used to examine the relationship between psychosocial problems, social support, self-management preferences, patients’ background factors and patients’ satisfaction with GP communication. The method of analysis allowed to include multiple parameters associated with patient satisfaction into the same statistical model. Previous studies have examined the relationship between some of the variables and satisfaction with GP communication separately but have not included them in one statistical model. Furthermore, the study included parameters, such as health status and perceived social support, that were found to be associated with general patient satisfaction with care^25 27 29^ but have not been examined with regards to patient satisfaction to GP communication. As general satisfaction of patients is related to satisfaction with the quality of doctor–patient communication,^59^ the relationships are not surprising. It needs to be kept in mind that the relationship between psychosocial problems, the encounter and satisfaction is complex and interpretation should be made with caution. A recent study by Gulbrandsen and colleagues showed, for example, that patient evaluations in a hospital setting are dynamic and that different variables play a role in first and later visits.^60^

There are several limitations to the study. First, the study used a cross-sectional design, so no causal relationships can be determined. Second, the data collection took place during the COVID-19 pandemic. Strict hygiene concepts, precautionary measures and infrastructural adaptation may have influenced participants’ participation in the study. Third, due to the COVID-19 lockdown, it was not possible to assess the total number of patients who frequented the GP practices. We therefore had to calculate the participants’ response rate from public databases. Fourth, the income was not reported by many patients (missing for n=197) and could therefore not be included in the model. Some other variables, such as chronic stress (n=125) and satisfaction (n=65), also had a relatively high number of missing values, indicating that participants did not always fill in the questionnaires thoroughly. It is possible that participants did not feel comfortable to fill in certain information, such as household income, or that they did not provide the correct information with regards to age and gender, for example. As the data collection was anonymous, we had no way of controlling this. Hence, the interpretation of the findings must be done with caution. Fifth, we could not check for double responses by participants who visited the facility several times within the data collection period as the participation was anonymous. However, double responses are unlikely as patients did usually not come in more than once during the length of the sampling period in the COVID-19 pandemic. Sixth, the assumption of this analysis was that the variables were missing at random. This implies that the missing mechanism may depend on other variables observed values, which is more realistic than the missing completely at random assumption. There seems to be no evidence that the missing mechanism could depend on non-available information besides the observed values. Finally, the sample is not representative of the German general population, particularly with regards to gender (the sample has a higher percentage of women than the general German population) and age (the sample is older than the general German population). This is not surprising in a sample of primary care patients as younger and healthier people go to the doctor less frequently. However, the higher occurrence of psychosocial problems may be (partly) attributed to the differences in age and/or gender. Hence, the effect may be overestimated due to the bias in the sample. We still believe that the study is making a valuable contribution as the authors are not aware of a better data set on German primary care patients.

The findings have several implications for GP practice: First, the study highlights the number of patients with psychosocial problems in primary care and that GPs may still underestimate the presence of problems in their patients. A routine screening could make sure that psychosocial problems are detected and may be considered during the consultation. Second, the study shows that not all patients with psychosocial problems would like support from their GP. Therefore, GPs should be aware of patients’ current social support and self-management preferences. Asking patients whether they currently have someone to support them may be crucial in supporting those in need. Finally, patients were generally satisfied with their GP’s communication, indicating that physician–patient communication works well in most cases.

More research is needed to better understand the prevalence of psychosocial problems in primary care. For example, it would be helpful to assess the prevalence of psychosocial problems from a GP’s and patient’s perspective in a representative sample. Furthermore, qualitative research is necessary to identify how physicians would like to be supported with psychosocial problems.

## Supplementary material

10.1136/bmjopen-2024-095489 online supplemental file 1

10.1136/bmjopen-2024-095489 online supplemental file 2

## Data Availability

Data are available upon reasonable request.
