# Supplementary material for: Relationship between psychosocial problems and satisfaction with GP communication in German primary care practices: a structural equation model based on the cross-sectional GPCare-1 patient study
Source: BMJ Open. 2025 May 7;15(5):e095489. doi: 10.1136/bmjopen-2024-095489 (PMC12060890; doi:10.1136/bmjopen-2024-095489)
Supplement: online supplemental file 1 [file bmjopen-15-5-s001.pdf]

Supplemental material 1

**About you:**					
**1. Age** __________ in years				**4. What is your current professional situation?**	
**2. Gender**	O Female	O Male	O Diverse	O Blue- or white-collar worker	O In training
**3. What is the highest level of education you have completed?**				O Self-employed	O Civil servant
O None		O University degree		O Retired / in pension	O Housewife/ -husband
O Secondary school up to 9th grade		O Vocational school		O Unemployed	O Study/internship/other
O Secondary school up to 10th grade		O Other		**5. In which industrial sector are you currently working, or have you been active last?**	
O Highschool (A-levels)				____	

**About your living situation:**			
**6. Are you married or in a relationship**	O No	O Yes	
**7. Are you currently taking care of people who are ill or in need of care?**	O No	O Yes	
**8. Are you or one of your parents not born in Germany?** *(multiple answers possible)*	O No	O Only me	O one or both of my parents
**9. How many people are living in your household (including yourself)** _____________ people			
**10. How high is the monthly net income of your household (approx. in euro)?** _____________ €			

**11. How many people are so close to you that you can count on them if you have great personal problems?**				
O None	O 1-2	O 3-5	O more than 5	
**12. How much interest and concern do people show in what you do?**				
O A lot	O Some	O Uncertain	O Little	O None
**13. How easy is it to get practical help from neighbours if you should need it?**				
O Very easy	O Easy	O Possible	O Difficult	O Very difficult

**About your health:**					
**14. I am patient of this general practitioner since**		O Less than 1 year	O 1-2 years	O 3-5 years	O More than 5 years
**15. How would you describe your general health status during the last 4 weeks?**					
O Excellent	O Very good	O Good	O moderate	O Bad	O Very bad
**16. Which of the following health problems apply to you?** *(multiple answers possible)*					
O Coronary artery disease (CAD)		O Diabetes		O Anxiety	
O Stroke		O Depression		O Chronic obstructive pulmonary disease (COPD)	
O High blood pressure		O Migraine			
O Back/ joint complains		O Sleeping disorder		O Other diseases	O None
**17. Over the past two weeks, how often have you been bothered by any of the following problems?**					
		Not at all	Several days	More than one half the days	Nearly every day
Little interest or pleasure in doing things		O	O	O	O
Feeling down, depressed, or hopeless		O	O	O	O

Copyright: Institute for Family Medicine and General Practice, University Hospital Bonn, Germany

Experiences and situations from everyday life:

18. How often have you experienced the following situations or feelings <u>during the last 3 months</u>?

	Never	Rarely	Sometimes	Often	Very Often
I feared that something unpleasant might happen	O	O	O	O	O
There were times I could not supress my worries	O	O	O	O	O
I tried, to no avail, to get appreciation through excellent performance	O	O	O	O	O
There were times I felt overwhelmed by worries	O	O	O	O	O
Although I did my best, my work was not valued	O	O	O	O	O
There were times I was unable to meet others´ expectations	O	O	O	O	O
There were times I could not stop worrying	O	O	O	O	O
There were times I had too many obligations	O	O	O	O	O
There were times I felt overwhelmed with work	O	O	O	O	O
I worried that I would not be able to accomplish my work	O	O	O	O	O
I felt that everything I had to deal with became too much	O	O	O	O	O
There were times I felt burdened by the responsibility for others	O	O	O	O	O

19. Which of the situations have you ever experienced? Which situation currently burdens you (no matter when it occurred)?

	Have I experienced		If yes: (still) burdens me
	No	Yes	
Excessive stress at work	O	O	O
Loss of job/unemployment	O	O	O
Feeling of loneliness	O	O	O
Taking care of a relative or (family) friend	O	O	O
Financial problems/debts that are difficult to negotiate	O	O	O
Death of a partner	O	O	O
Physical attacks (e.g. being beaten or slapped, ...)	O	O	O
Psychological damaging actions or threats (e.g. being insulted, threatened, harassed or pressured, ...)	O	O	O
Sexual harassment (e.g. salacious remarks, unwanted touching, ...)	O	O	O
Sexual assaults (e.g. physical sexual violence, sexual abuse, …)	O	O	O

20. Please indicate the extent to which you agree with the following statements. Think of the personal strain mentioned in question 19.

I	Totally agree	Agree	Neither agree nor disagree	Disagree	Totally agree
My doctor asks me about stress caused by personal strains	O	O	O	O	O
My doctor gives me enough space to describe personal strains	O	O	O	O	O
My doctor makes me feel comfortable talking about sensitive things	O	O	O	O	O
I get the feeling that my doctor takes my problems very seriously	O	O	O	O	O
I rather overcome personal strain without help from my doctor	O	O	O	O	O
Discussing personal strains with my doctor makes me uncomfortable	O	O	O	O	O
I would prefer my doctor to ask me directly about personal strains	O	O	O	O	O
I would prefer the doctor to give me a questionnaire regarding my personal strains	O	O	O	O	O

Copyright: Institute for Family Medicine and General Practice, University Hospital Bonn, Germany
