# Supplementary material for: Relationship between psychosocial problems and satisfaction with GP communication in German primary care practices: a structural equation model based on the cross-sectional GPCare-1 patient study
Source: BMJ Open. 2025 May 7;15(5):e095489. doi: 10.1136/bmjopen-2024-095489 (PMC12060890; doi:10.1136/bmjopen-2024-095489)
Supplement: online supplemental file 2 [file bmjopen-15-5-s002.pdf]

Supplemental material 2

Cluster analysis

Methods. To combine clustering with multiple imputation, we applied a clustering to our data following the approach by Lee et al. [1]. In particular, k-medoids clustering [2] was used. Medoids are a more robust version than k-means and these are easier to interpret, because medoids correspond to observed data points. Mixed measurements variables (for example ratio scale age and ordinal questions related to communication) were considered by using Gower distance matrix [3]. For each multiple imputed data, set cluster sizes from 2 to 28 were evaluated and the best cluster size was chosen according to the highest average silhouette width [4].

Results. The cluster analysis identified seven final clusters, with their medoid values and the distribution of observed variables. Table 1 presents an overview of the main variables used in our analyses across the seven identified clusters.
The mean average silhouette width over the multiple imputed data sets was 0.20337, which falls below the threshold of 0.25. This indicates that the identified clusters provide only weak support for unobserved subgroup heterogeneity in the data to validate the relevance of the cluster assignments, a pooled likelihood ratio test [5] was conducted. This test compared the original structural equation model with an extended version that included the final clusters as a covariate. The resulting p-value of 0.285 suggests that incorporating the cluster information did not systematically improve model fit.

Variable / Cluster	1	2	3	4	5	6	7
Personal strains (1 < 2 < 3 < 4 < 5)	3	5	3	3	4	4	4
Enough space (1 < 2 < 3 < 4 < 5)	3	5	3	3	4	4	5
Feel comfortable (1 < 2 < 3 < 4 < 5)	3	5	4	3	4	4	5
Problems seriously (1 < 2 < 3 < 4 < 5)	4	5	4	3	4	5	5
Self-Management (1 < 2 < 3 < 4 < 5)	3	3	4	4	4	3	3
Age (years)	48	48	56	53	47	58	44
Gender (0 = female, 1= male)	0	0	1	1	0	1	0
Social support (sum score)	9	10	11	11	12	9	11
Years with physician	4	4	4	4	4	4	4
Depressive symptoms (sum score)	2	1	1	1	2	2	2
Chronic stress (sum score)	21	12	16	12	20	13	19
Psychosocial problems (sum score)	0	0	0	0	0	0	0
Health status (1 < 2 < 3 < 4 < 5)	3	3	4	3	3	3	3

Table 1: Cluster medoids after multiple imputation. Likertscale items with five categories correspond to values „do not agree at all“ (1), „tend to disagree“ (2), „agree partly“ (3), „tend to agree“ (4) and „fully agree“ (5).

Discussion. These findings suggest that the identified clusters do not provide substantial discriminatory power regarding the underlying data structure. This may be

explained by high similarity among subgroups, suggesting that GP practices may have had a similar variety of patients. As the findings indicate that the clusters do not significantly improve the analyses performed without clustering, they were not considered in our analyses.

Complete case analysis

Methods. A complete case analysis was conducted. The complete case data consisted of 67.32 % of all GP in the original data. It was tested whether the coefficients of the observed model were equal to the coefficients observed in the imputed model. Then, the differences between each model`s regression coefficients of social support, self-management, age, health status and impairment were compared with a two-group Z-test. This approach took both the standard deviations of the complete case and the multiple imputed structural equation model into account.

Results. Both models were found to share the same covariates with p-values smaller then 0.05 with the only exception impairment. The measurement model coefficients of variables “enough space” (p-value 0.9615), “feel comfortable” (p-value 0.7599) and “Problems seriously” (p-value 0.7799), social support (p-value 0.6172), self-management (p-value 0.2804), age (p-value 0.7350), health status (p-value 0.7813) and impairment (p-value 0.2840) were not systematically different between the two models. Figure 3 shows the results of the complete case analysis. The findings indicate that there is no indication of systematic differences between the complete case structural equation model and the multiple imputed version.

Figure 3. Complete case analysis

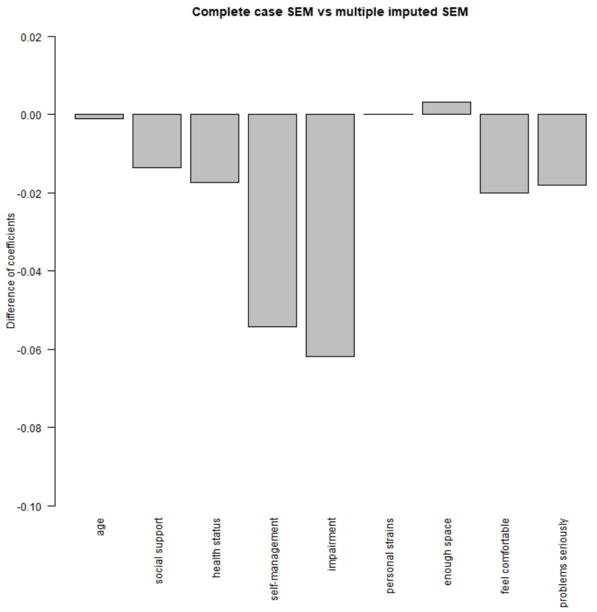

Discussion. The two models did not show different p-values, indicating that the multiple imputed model is a good fit for the data and that imputation of missing values did not change the model. Only one variable was significant in the complete case model and not in the multiple imputed model (impairment). However, the estimates of the multiple imputed model include more information. Therefore, we think the effect of

impairment in the complete case model could be the result of the smaller sample size and should be interpreted with caution.

Literature

1 Lee, J. W., & Harel, O., (2022), Incomplete clustering analysis via multiple imputation, Journal of Applied Statistics, 50(9), Pages 1962–1979, https://doi.org/10.1080/02664763.2022.2060952
2 Kaufman, L. and Rousseeuw, P.J. (1990), Partitioning Around Medoids (Program PAM), In Finding Groups in Data (eds L. Kaufman and P.J. Rousseeuw), https://doi.org/10.1002/9780470316801.ch2
3 Gower, J. C., (1971), A General Coefficient of Similarity and Some of Its Properties, Biometrics, 27(4), 857–871, https://doi.org/10.2307/2528823
4 Rousseeuw, P. J., (1987), Silhouettes: A graphical aid to the interpretation and validation of cluster analysis, Journal of Computational and Applied Mathematics, Journal of Computational and Applied Mathematics, 20, 53-65, https://doi.org/10.1016/0377-0427(87)90125-7
5 Li, K.-H., Meng, X.-L., Raghunathan, T. E., & Rubin, D. B., (1991), Significance levels from repeated p-values with multiply-imputed data, Statistica Sinica, 1(1), 65–92, https://www.jstor.org/stable/24303994
