# Supplementary material for: Relationship between psychosocial problems and satisfaction with GP communication in German primary care practices: a structural equation model based on the cross-sectional GPCare-1 patient study
Source: BMJ Open. 2025 May 7;15(5):e095489. doi: 10.1136/bmjopen-2024-095489 (PMC12060890; doi:10.1136/bmjopen-2024-095489)
Supplement: Supplementary file 3 [file bmjopen-2024-095489.reviewer_comments.pdf]

PEER REVIEW HISTORY

BMJ Open publishes all reviews undertaken for accepted manuscripts. Reviewers are asked to complete a checklist review form and are provided with free text boxes to elaborate on their assessment. These free text comments are reproduced below.

ARTICLE DETAILS

Title (Provisional)

Relationship between psychosocial problems and satisfaction with GP communication in German primary care practices: A structural equation model based on the cross-sectional GPCare-1 patient study

Authors

Sachschal, Juliane; Welchowski, Thomas; Offenberg, Luisa; Oberholz, Maja; Gavrilov, Boris; Ikar, Nur; Hunzelar, Carmen; Bockheim, Florian; Paños-Willuhn, Joana; Weltermann, Birgitta

---

VERSION 1 - REVIEW

**Reviewer**	**1**
**Name**	**Gulbrandsen, Pal**
**Affiliation**	**Universitetet i Oslo Det medisinske fakultet**
**Date**	**15-Nov-2024**
**COI**	**None**

The authors have adequately responded to my first review, Because two of the reviewers (incl me) asked for a more precise description of the SEM, I think it would be good to have a statistician overview the model, but this is for the editor to decide.

There are still a few minor things that need amendment, most of them are editorial.

1. I think the lines 47-50 in the abstract are not perfectly aligned with line 308-311 in the results section and lines 320-324 in the discussion. in the abstract I miss 'lower' or 'higher' before 'age'. Also, because these results are presented with outcome lower satisfaction (abstract), and higher satisfaction (discussion), one gets easily confused. In the abstract it says 'perceived social support', in the results only 'social support' or 'less social support', and 'higher social support' in the discussion, this adds to the confusion. I advise to avoid redundancy (results and discussion), and be exact in the reporting of what affects what in which direction.

Minor issues:

2. In line 87 what looks like references 60-61are good, but isn't it odd not to re-order references in the sequence they appear in the text?

3. In line 91 I miss a reference, I would have used reference 61.

4. In lines 209-211 they refer to figure 3, even if figures 1 and 2 have not been presented yet. Could this be fixed? By the way, my personal view is that figure 1 would better be represented

by a table (more precise presentation), regarding figure 2 I am inclined to suggest the same solution.

5. My name is wrongly written in lines 339 and 361.

6. In lines 361-2 the findings in our study are somewhat imprecisely presented: It was not the number of encounters that influenced the evaluation, but we observed a distinction between patient evaluations made in first visits with a doctor, compared to later visits. This was in hospital practice, where there are many first visits. I don't know how that is in German general practice.

7. In Table 1, line 'male', a % sign is superfluous.

8. To avoid confusion with references, I would suggest that the missing values in brackets might better be marked with an asterisk?

I enjoy that this research groups has addressed the importance of people's lives in medical practice. I think maybe the last section of the discussion might have gained on mentioning the insights of philosophers like Hegel, Husserl, and/or Merleau-Ponty - point is: when will medicine stop seeing patients just as bodies or minds, rather than persons interwoven with the world throughout their lives? But this is not a requirement, rather a suggestion to open new horizons to the reader.

**Reviewer**	**2**
**Name**	**van den Berg, Joost**
**Affiliation**	**Amsterdam University Medical Centres, Internal Medicine**
**Date**	**02-Dec-2024**
**COI**	**None**

I am very happy to read that authors addressed the reviewers concerns so thoroughly. I feel that all previous concerns have been addressed and I found no issues in the review checklist.

Some minor issues that I feel remain:

The answer on the desired sample size in the comments to the reviewers does not seem to have been incorporated in the manuscript. Rather, the authors only describe the time frame during which the study was conducted.

**Reviewer**	**3**
**Name**	**Kelson, Zoe**
**Affiliation**	**University of Exeter, Mathematics**
**Date**	**14-Feb-2025**
**COI**	**None**

This study examined the relationship between primary care patients´ psychosocial problems, other patient characteristics that are associated with satisfaction with overall care, and satisfaction with GP communication.

Reviewer comments:

"A total of 813 patients from 12 GP practices participated." [Abstract]

and

"The study was conducted in 12 primary care teaching practices, which are affiliated with the Institute of General Practice and Family Medicine, University of Bonn and University Hospital Bonn. Practices were selected based on different socio demographic regional characteristics (e.g., age structure, population density, proportion of migrants) to ensure a coverage of differing population groups. "

and

"As practices were too busy during the Covid 19-pandemic, the response rate was calculated based on average data of patients per practice from a public German data base [31] and a documented 40% reduction in patient volume in GP practices [32]. The calculated response rate was 24.1%."

and

"The GPCare-1 data set included 813 adult GP patients. Characteristics are displayed in Table 1. "

The authors satisfactorily comment on the cohort representativeness in the discussion of the study limitations.

Can the authors please consider exploring differences and clustering by GP practice?

"The survey was conducted in summer 2020 during a Covid-19 lockdown." [Abstract]

and

"The survey was conducted between June and August 2020 which happened to be during the second Covid-19 lockdown in Germany."

The authors transparently acknowledge this limitation in the article, and provide a discussion on this in the study limitations.

"The GPCare-1 questionnaire comprised a total of 48 questions. "

Can a copy of this questionnaire please be provided?

"Patients filled an anonymous 2-page questionnaire on various socio-demographic, medical characteristics, and their satisfaction with GP communication." [Abstract]

The authors address the risk of self-reporting bias and missing data in the discussion of the study limitations.

"Differences in prevalence were investigated using Chi-square test."

A suitable statistical test has been applied here.

"Missing rates are displayed in brackets behind the respective item"

and

"Table 1: Sample description, N=813. Missing values are described after each variable [N]. "

Can the authors please further comment on whether this missing data can be considered to be missing at random?

"Structural equation modelling evaluated associations of various patient characteristics including psychosocial problems with GP communication" [Abstract]

and

"Associations between communication and satisfaction as well as their dependencies were jointly estimated by a structural equation model (SEM). The latent continuous endogenous variable satisfaction with GP communication represents one dimension of patient satisfaction with quality of care, including perceived consideration for the patient [41] and emotional support [38]. Communication quality is a sub-dimension of the interpersonal qualities of a general practitioner [42]. The SEM consists of a structural component that is represented on the left side of the latent variable in Figure 3 and a measurement model displayed on the right side of the latent variable."

and

"The structural component part can be interpreted analogously to a linear regression framework [58]. All observed items on the left side (age, gender, social support, time with the GP, depression score, chronic stress, number of current psychosocial problems, health status and communication preference) correspond to independent covariates and the latent variable satisfaction with GP communication is the dependent response variable. The latent variable is assumed to be continuous and normal distributed conditional on the items"

An appropriate methodology has been applied, which is clearly described by the authors.

"Missing values were imputed by multiple imputation by chained equations [43] with 25 iterations and repetitions. Continuous covariates (e. g. age, Oslo score) were imputed by predictive mean matching, nominal covariates (e. g. gender) were imputed by multinomial regression [44] and ordinal covariates (e. g. health status) were imputed by proportional odds models [45]. For each multiple imputed data set, a structural equation model was estimated "

The authors have applied a rigorous methodology for handling missing data.

Can the authors please consider conducting a sensitivity analysis on this approach (e.g. a complete-case analysis) to help demonstrate the robustness of the study findings to missing data?

"Norman [47] points out that many previous studies show the robustness of Likert scales to parametric assumption violations and in case of five point Likert scales, a simulation study showed that t-test and Mann Whitney-Wilcoxon Tests had similar power [48]."

Can the authors please clarify which statistical tests they applied here (i.e. t-test or Mann Whitney)?

"According to recommendations of Kline [46] we report several pooled structural equation model goodness of fit statistics. "

Can the authors please specify the performance metrics that they applied here?

"The sample size to estimated parameters ratio is 47.71 which is more than double than the recommended minimum ratio of 20 in Kline [40]."

The authors demonstrate the robustness of the sample size.

"The patient sample is not representative but in some characteristics similar to national comparative values • The study used a cross-sectional design so no causal relationships can be determined." [strengths and limitations of this study]

and

"There are several limitations to the study. Firstly, the study used a cross-sectional design so no causal relationships can be determined. Secondly, the data collection took place during the Covid-19 pandemic. Strict hygiene concepts, precautionary measures, and infrastructural adaptation may have influenced participants' participation in the study. Thirdly, due to the Covid-19 lockdown, it was not possible to assess the total number of patients that frequented the GP practices. We therefore had to calculate the participants' response rate from public databases. Fourthly, the income was not reported by many patients (missing for n = 197) and could therefore not be included in the model. Some other variables, such as chronic stress (n = 125) and satisfaction (n = 65) had also a relatively high number of missing values, indicating that participants did not always fill in the questionnaires thoroughly. It is possible that participants did not feel comfortable to fill in certain information, such as household income, or that they did not provide the correct information with regards to age and gender, for example. As the data collection was anonymous, we had no way of controlling this. Hence, the interpretation of the findings must be done with caution. Fifthly, we could not check for double responses by participants who visited the facility several times within the data collection period, as the participation was anonymous. However, double responses are unlikely, as patients did usually not come in more than once during the length of the sampling period in the Covid-19 pandemic. Finally, the sample is not representative of the German general population, particularly with regards to gender (the sample has a higher percentage of women than the General German population) and age (the sample is older than the General German population). This is not surprising in a sample of primary care patients as younger and healthier people go to the doctor less frequently. However, the higher prevalence for psychosocial problems may be (partly) attributed to the differences in age and / or gender. Hence, the effect may be overestimated due to the bias in the sample. We still believe that the study is making a valuable contribution, as the authors are not aware of a better dataset on German primary care patients."

The authors provide a thorough discussion on the study limitations.

Thanks for providing a copy of the STROBE checklist.

VERSION 1 - AUTHOR RESPONSE

Response to Reviewers

Reviewer: 1
Dr. Pal Gulbrandsen, Universitetet i Oslo Det medisinske fakultet

Comments to the Author:
The authors have adequately responded to my first review, Because two of the reviewers (incl me) asked for a more precise description of the SEM, I think it would be good to have a statistician overview the model, but this is for the editor to decide.

Thank you very much for this feedback. We are pleased to hear that you are happy with our responses to your first comments. We would also like to thank you for taking the time again to give us some additional feedback on our revised manuscript.

Please note that we refer to the marked copy of the manuscript in our responses.

There are still a few minor things that need amendment, most of them are editorial.

1. *I think the lines 47-50 in the abstract are not perfectly aligned with line 308-311 in the results section and lines 320-324 in the discussion. in the abstract I miss 'lower' or 'higher' before 'age'. Also, because these results are presented with outcome lower satisfaction (abstract), and higher satisfaction (discussion), one gets easily confused. In the abstract it says 'perceived social support', in the results only 'social support' or 'less social support', and 'higher social support' in the discussion, this adds to the confusion. I advise to avoid redundancy (results and discussion), and be exact in the reporting of what affects what in which direction.*

Thank you for making us aware of these reporting problems. We have corrected these (in the abstract and results section, l. 47-49 and l. 337-339) and made sure that they align with the discussion section. Specifically, we changed the reporting so that we generally refer to which variables contribute to higher satisfaction with GP communication, whereas before we sometimes reported to more or less satisfaction, which may have resulted in seemingly contradicting statements. We hope this can resolve any potential confusion to the reader.

These are the sentences in the manuscript:

Abstract: Higher social support, preference to solve one’s problem without GP help, higher age, and better health status predicted more satisfaction with physician-patient-communication […].

Results section: The variables social support, health status and self-management preference, and age predicted the latent variable satisfaction, suggesting that higher age, more social support, better health status and the preference to not solve problems on their own was associated with higher satisfaction with GP communication.

Discussion: Higher social support, preference to solve one’s problem without GP help, higher age, and better health status but not the number of psychosocial problems predicted more satisfaction with physician-patient-communication.

###

Minor issues:

2. In line 87 what looks like references 60-61are good, but isn't it odd not to re-order references in the sequence they appear in the text?

We have corrected this mistake.

##

3. In line 91 I miss a reference, I would have used reference 61.

We agree that this sentence would benefit from a reference. We have added the suggested reference, which is now reference 15 (l. 99).

###

4. In lines 209-211 they refer to figure 3, even if figures 1 and 2 have not been presented yet. Could this be fixed? By the way, my personal view is that figure 1 would better be represented by a table (more precise presentation), regarding figure 2 am inclined to suggest the same solution.

We have fixed the first problem with regards to Figure 3 (l.315), which is now Figure 1. We have replaced Figure 1 with Table 2 for a more precise presentation, as suggested.
However, we would prefer to keep Figure 2 as a figure as we find it easier for the reader to read the results with the visual support of a figure.

###

5. My name is wrongly written in lines 339 and 361.

My sincere apologies for this. We have corrected this mistake.

##

6. In lines 361-2 the findings in our study are somewhat imprecisely presented: It was not the number of encounters that influenced the evaluation, but we observed a distinction between patient evaluations made in first visits with a doctor, compared to later visits. This was in hospital practice, where there are many first visits. I don't know how that is in German general practice.

Thank you for making us aware of this imprecision. We have corrected this mistake and hope that it is to your satisfaction (l. 385-388). The new sentence is:

“A recent study by Gulbrandsen and colleagues showed, for example, that patient evaluations in a hospital setting are dynamic, and that different variables play a role in first and later visits [56].”

###

7. In Table 1, line 'male', a % sign is superfluous.

We have corrected this mistake.

##

8. To avoid confusion with references, I would suggest that the missing values in brackets might better be marked with an asterisk?

We have updated Table 1 accordingly.

##

I enjoy that this research groups has addressed the importance of people's lives in medical practice. I think maybe the last section of the discussion might have gained on mentioning the insights of philosophers like Hegel, Husserl, and/or Merleau-Ponty - point is: when will medicine stop seeing patients just as bodies or minds, rather than persons interwoven with the world throughout their lives? But this is not a requirement, rather a suggestion to open new horizons to the reader.

Thank you for that interesting suggestion. The mind body problem has a long-standing history in philosophy. We agree that neither the mind or body can exist independently of the other and science should take this into account to describe the whole phenomenon. This work captures subjective measurements and we believe that those provide meaningful extra information besides other more objective measurements like biomarkers. However, none of the authors feels that their understanding of the philosophy of Hegel, Husserl or Merleau-Ponty is sufficiently deep to discuss these philosophical questions as part of the manuscript.

##

##

Reviewer: 2
Dr. Joost van den Berg, Amsterdam University Medical Centres
Comments to the Author:
I am very happy to read that authors addressed the reviewers concerns so thoroughly. I feel that all previous concerns have been addressed and I found no issues in the review checklist.

Thank you very much for this feedback. We would also like to thank you to taking the time again to read our manuscript.

Some minor issues that I feel remain:
The answer on the desired sample size in the comments to the reviewers does not seem to have been incorporated in the manuscript. Rather, the authors only describe the time frame during which the study was conducted.

Thank you very much for this comment.

a) <u>Was a power analysis conducted to establish the desired sample size</u>? We did not conduct an explicit power analysis for the structural equation model. However, we made sure that the sample size is adequate according to current recommendations in the literature. The sample size justification depends on properties of the data generating process and goals of the analysis. For example, with the multivariate normal assumption, sample sizes between 200 – 400 are sufficient to avoid convergence problems and estimation bias (Jackson, 2001) and another article suggested a ratio of sample size to number of parameters in the model of at least 10 (Bentler, 1987). This ratio for the observed data 47.70 is much larger than the recommended value. Furthermore, Hancock (2013) investigated application of power analysis for goodness of fit statistics such as the root mean square error of approximation.

For example, assuming a structural equation model with 15 degrees of freedom, medium amount of model misspecification, significance level 0.05 and a power 0.8 yielded a sample size of 741 in the previously mentioned book. This sample size requirement was also surpassed in this study with 811 observations. In summary, the sample size of this study is adequate according to these recommendations.

This is described in line 281ff:

"The sample size to estimated parameters ratio is 47.71 which is more than double than the recommended minimum ratio of 20 in Kline [40]."

b) Why was this specific time period chosen? The specific time period was chosen to reach a sample size of 1000 participants, assuring to also detect psychosocial problems with a low prevalence based on the prevalence rates found in the DEGS1 study. This is also registered in our trial registration: https://drks.de/search/en/trial/DRKS00022330/details

The reported time period was a result of the time frame that was feasible under the extraordinary conditions in primary care practices during the Covid-19 pandemic lockdowns.

We have updated this in the methods section (l. 150ff):

“The targeted sample size was 1000 patients. The goal could not be reached due to slower recruitment in primary care practices during the Covid-19 pandemic with its lockdowns.”

References

Jackson, D. L. (2001). Sample size and number of parameter estimates in maximum likelihood confirmatory factor analysis: A Monte Carlo investigation. Structural Equation Modeling, 8, 205–223.

Bentler, P. M., & Chou, C.-P. (1987). Practical issues in structural equation modeling. Sociological Methods and Research, 16, 78–117.

Hancock, G.R. (2013). Power analysis in structural equation modeling. In G.R. Hancock & R.O. Mueller (Eds.), Structural Equation Modeling: A second Course, 2nd Edition (pp.117 - 162). Charlotte, NC: Information Age Publishing.

##

##

Reviewer: 3
Prof. Zoe Kelson, University of Exeter Comments
to the Author:
This study examined the relationship between primary care patients´ psychosocial problems, other patient characteristics that are associated with satisfaction with overall care, and satisfaction with GP communication.

Thank you very much for taking the time to read our manuscript and provide detailed feedback to the statistical methods of our research. We very much appreciate your comments and value your insights to improve our manuscript.

Please note that we refer to the marked copy of the manuscript when we refer to lines in our responses.

Reviewer comments:

"A total of 813 patients from 12 GP practices participated." [Abstract]
and
"The study was conducted in 12 primary care teaching practices, which are affiliated with the Institute of General Practice and Family Medicine, University of Bonn and University Hospital Bonn. Practices were selected based on different socio demographic regional characteristics (e.g., age structure, population density, proportion of migrants) to ensure a coverage of differing population groups. "
and
"As practices were too busy during the Covid 19-pandemic, the response rate was calculated based on average data of patients per practice from a public German data base [31] and a documented 40% reduction in patient volume in GP practices [32]. The calculated response rate was 24.1%."
and
"The GPCare-1 data set included 813 adult GP patients. Characteristics are displayed in Table 1. "
The authors satisfactorily comment on the cohort representativeness in the discussion of the study limitations.

Thank you, we are happy to hear that.

##

Can the authors please consider exploring differences and clustering by GP practice?

We followed your suggestion and applied clustering to our data. We decided to follow the approach by Lee et al. (2022) to combine clustering with multiple imputation. In particular, k-medoids clustering (Kaufman, 1990) was used. Medoids are a more robust version than k-means and these are easier to interpret, because medoids correspond to observed data points. Mixed measurements variables (for example ratio scale age and ordinal questions related to communication) were taken into account by using Gower distance matrix (Gower, 1971). For each multiple imputed data set cluster sizes from 2 to 28 were evaluated and the best cluster size was chosen according to the highest average silhouette width (Rousseeuw, 1987).

The mean average silhouette width over the multiple imputed data sets was 0.20337. Average silhouette widths below 0.25 indicate only weak support for non-observed subgroup heterogeneity in the data. A pooled likelihood ratio test (Li, 1991) of the original structural equation model and the same model with additional covariate containing final clusters gave a p-value of 0.285. This confirms that the cluster information does not systematically improve the fit of the structural equation model to the data. For reference, the medoids of the final seven clusters were included as additional supplementary material.

The following sentences were added to the methods section (l. 267ff):

“We further conducted several sensitivity analyses (cluster analysis, complete case analysis). The cluster information did not systematically improve the fit of the structural equation model to the data. (…). Results of both analyses can be found in the supplemental material.”

##

"The survey was conducted in summer 2020 during a Covid-19 lockdown." [Abstract] and "The survey was conducted between June and August 2020 which happened to be during the second Covid-19 lockdown in Germany."
The authors transparently acknowledge this limitation in the article, and provide a discussion on this in the study limitations.

We are happy to hear that.
###

"The GPCare-1 questionnaire comprised a total of 48 questions. " Can a copy of this questionnaire please be provided?

We have attached all the questions used at the end of this response.
###

"Patients filled an anonymous 2-page questionnaire on various socio-demographic, medical characteristics, and their satisfaction with GP communication." [Abstract]
The authors address the risk of self-reporting bias and missing data in the discussion of the study limitations.

Thank you, we are happy to hear that.

###

"Differences in prevalence were investigated using Chi-square test." A suitable statistical test has been applied here.

Thank you, we are happy to hear that.

###

"Missing rates are displayed in brackets behind the respective item"
and
"Table 1: Sample description, N=813. Missing values are described after each variable [N]. "

Can the authors please further comment on whether this missing data can be considered to be missing at random?

This is a good point, thank you. The assumption of this analysis was that the variables were missing at random. That means that the missing mechanism may depend on other variables observed values, which is more realistic than the missing completely at random assumption. There seems to be no evidence, that the missing mechanism could depend on non-available information besides the observed values.

The following statement was added into the Section discussion as potential limitation (ll. 414ff).

“Sixthly, the assumption of this analysis was that the variables were missing at random. This implies that the missing mechanism may depend on other variables’ observed values. Such a scenario is more realistic than the assumption of data missing completely at random. There

is no evidence that the missing mechanism could depend on non-available information besides the observed values.”

##

"Structural equation modelling evaluated associations of various patient characteristics including psychosocial problems with GP communication" [Abstract]
and
"Associations between communication and satisfaction as well as their dependencies were jointly estimated by a structural equation model (SEM). The latent continuous endogenous variable satisfaction with GP communication represents one dimension of patient satisfaction with quality of care, including perceived consideration for the patient [41] and emotional support [38]. Communication quality is a sub-dimension of the interpersonal qualities of a general practitioner [42]. The SEM consists of a structural component that is represented on the left side of the latent variable in Figure 3 and a measurement model displayed on the right side of the latent variable."
and
"The structural component part can be interpreted analogously to a linear regression framework [58]. All observed items on the left side (age, gender, social support, time with the GP, depression score, chronic stress, number of current psychosocial problems, health status and communication preference) correspond to independent covariates and the latent variable satisfaction with GP communication is the dependent response variable. The latent variable is assumed to be continuous and normal distributed conditional on the items"
An appropriate methodology has been applied, which is clearly described by the authors.

Thank you very much, we are happy to hear that.
##

"Missing values were imputed by multiple imputation by chained equations [43] with 25 iterations and repetitions. Continuous covariates (e. g. age, Oslo score) were imputed by predictive mean matching, nominal covariates (e. g. gender) were imputed by multinomial regression [44] and ordinal covariates (e. g. health status) were imputed by proportional odds models [45]. For each multiple imputed data set, a structural equation model was estimated "
The authors have applied a rigorous methodology for handling missing data.

Can the authors please consider conducting a sensitivity analysis on this approach (e.g. a complete-case analysis) to help demonstrate the robustness of the study findings to missing data?

In addition to the sensitivity analysis with clustering, a complete case analysis was conducted. The complete case data consisted of 67.32 % of all observation points in the original data. The differences between each models’ regression coefficients of social support (p-value 0.6172), self-management (p-value 0.2804), age (p-value 0.7350), health status (p- value 0.7813) and impairment (p-value 0.2840) were compared with a two-group Z-test. This approach took both the standard deviations of the complete case and the multiple imputed structural equation model into account. Differences of the measurement model coefficients were even smaller and “enough space” (p-value 0.9615), “feel comfortable” (p-value 0.7599) and “takes problems seriously” (p-value 0.7799) were not systematically different.

Thus, there is no indication of systematic differences between the complete case structural equation model and the multiple imputed version. Both models share the same covariates with p-values smaller then 0.05 with the only exception impairment. In this case, the complete case p-value was 0.0036. The estimates of the multiple imputed model include more information. Therefore, we think that the effect of impairment in the complete case model could be the result of the smaller sample size.

For reference, the bar-plot of the difference of the model coefficients and their Z-test p-values were added to the supplemental material.

The following sentence was added to the methods section (l. 267ff):

“We further conducted several sensitivity analyses (cluster analysis, complete case analysis). (…). The complete case analysis did not indicate systematic differences between the complete case structural equation model and the multiple imputed version. Results of both analyses can be found in the supplemental material.”

##

"Norman [47] points out that many previous studies show the robustness of Likert scales to parametric assumption violations and in case of five point Likert scales, a simulation study showed that t-test and Mann Whitney-Wilcoxon Tests had similar power [48]."
Can the authors please clarify which statistical tests they applied here (i.e. t-test or Mann Whitney)?

For the tests of the regression coefficients in the structural equation model we applied t-tests.

We are afraid that this sentence might have been unclear: The examples in this sentence were meant to show that Likert scales have been shown to be robust for the use of parametric tests rather than refer to the specific statistical tests that we used. We have corrected the sentence so that it’s meaning becomes clearer to the reader.

"Norman [47] points out that many previous studies show the robustness of Likert scales to parametric assumption violations and that parametric tests can be applied for Likert scales.”

##

"According to recommendations of Kline [46] we report several pooled structural equation model goodness of fit statistics. "
Can the authors please specify the performance metrics that they applied here?

The following text was added to the methods section:

… Kline [46] we report several pooled structural equation model goodness of fit statistics. Among those is the Chi-Square test statistic of the structural equation model, which is an omnibus test with null hypothesis that all coefficients are zero. The Bentler Comparative Fit Index [[1]] compares the model with the previous null hypothesis model and calculates the relative difference. Steiger-Lind Root Mean Square Error of Approximation [[51]] and the standardized root mean square residual both compare the estimated values of the structural equation model with the observed data. In the former values below 0.05 and for the latter measure values below 0.08 indicate a good model fit (Hair, 2021).

References

Hair, J. F., Hult, G. T. M., Ringle, C. M., Sarstedt, M., Danks, N.P., & Ray, S, (2021), Partial Least Squares Structural Equation Modeling (PLS-SEM) Using R, Springer

##

"The sample size to estimated parameters ratio is 47.71 which is more than double than the recommended minimum ratio of 20 in Kline [40]."
The authors demonstrate the robustness of the sample size.

We are happy to hear this.

##

"The patient sample is not representative but in some characteristics similar to national comparative values • The study used a cross-sectional design so no causal relationships can be determined." [strengths and limitations of this study]
and
"There are several limitations to the study. Firstly, the study used a cross-sectional design so no causal relationships can be determined. Secondly, the data collection took place during the Covid-19 pandemic. Strict hygiene concepts, precautionary measures, and infrastructural adaptation may have influenced participants' participation in the study. Thirdly, due to the Covid-19 lockdown, it was not possible to assess the total number of patients that frequented the GP practices. We therefore had to calculate the participants' response rate from public databases. Fourthly, the income was not reported by many patients (missing for n = 197) and could therefore not be included in the model. Some other variables, such as chronic stress (n = 125) and satisfaction (n = 65) had also a relatively high number of missing values, indicating that participants did not always fill in the questionnaires thoroughly. It is possible that participants did not feel comfortable to fill in certain information, such as household income, or that they did not provide the correct information with regards to age and gender, for example. As the data collection was anonymous, we had no way of controlling this. Hence, the interpretation of the findings must be done with caution. Fifthly, we could not check for double responses by participants who visited the facility several times within the data collection period, as the participation was anonymous. However, double responses are unlikely, as patients did usually not come in more than once during the length of the sampling period in the Covid-19 pandemic. Finally, the sample is not representative of the German general population, particularly with regards to gender (the sample has a higher percentage of women than the General German population) and age (the sample is older than the General German population). This is not surprising in a sample of primary care patients as younger and healthier people go to the doctor less frequently. However, the higher prevalence for psychosocial problems may be (partly) attributed to the differences in age and / or gender. Hence, the effect may be overestimated due to the bias in the sample. We still believe that the study is making a valuable contribution, as the authors are not aware of a better dataset on German primary care patients."
The authors provide a thorough discussion on the study limitations.

We are happy to hear this.

##
